# Application of the Healthy Eating Index in a multicultural population: introduction of Adaptive Component Scoring

**DOI:** 10.3389/fnut.2025.1511230

**Published:** 2025-02-05

**Authors:** David L. Katz, Lauren Q. Rhee, Dina L. Aronson

**Affiliations:** Diet ID/Tangelo (Intend, Inc.), Birmingham, MI, United States

**Keywords:** diet quality, dietary index, diet score, Healthy Eating Index, dietary patterns, multicultural diets, nutrition, food groups

## Abstract

The United States, and many modern nations, represent assemblies of many cultural groups. Such groups are often influenced, sometimes profoundly, by the culinary traditions of their countries of origin, resulting in a diversity of cultural dietary patterns. Such patterns all derive key elements of nutritional quality from essential food groups—such as vegetables and fruits—but vary in their inclusion of “discretionary” food groups, such as dairy. The application of robust, validated, and standardized diet quality scoring is important in nutrition research, and in the food-as-medicine movement at large if what is being “managed” is to be measured. While robustly validated, the Healthy Eating Index is closely aligned with the *Dietary Guidelines for Americans*, and thus may not readily account for all multicultural dietary variations. Other diet quality metrics account for deviation from the prevailing American dietary pattern, but none does so in a way that expressly adapts to food components included or excluded so that “credit” for nutritional quality is appropriately assigned in all cases using a standard metric. In this context, we introduce and explain Adaptive Component Scoring as applied to the Healthy Eating Index in the service of advancing fair and universal diet quality scoring. Implications for nutrition research and food-as-medicine initiatives are briefly enumerated.

## Introduction

The Healthy Eating Index (HEI) (1) and the related Alternative Healthy Eating Index (2) are among the most widely used and robustly validated measures of overall diet quality in the United States. These measures have been correlated directly with all-cause mortality and total chronic disease risk in large cohorts (3). Overall diet quality measured accordingly is now recognized as the single leading predictor variable for premature death in the United States (4), and much of the world (5).

Despite these strengths, there are important limitations to the HEI. The metric is closely aligned with the Dietary Guidelines for Americans (6), and accordingly confers credit for food groups that prevail in the American diet, including dairy, meat, poultry, fish, seafood, and grains. Whereas meat, poultry, and fish are assigned to a “protein” category in the HEI scoring construct, for which legumes may substitute, the omission of dairy or grains from a dietary pattern reduces the total, achievable HEI score.

Of note, an array of traditional East and Southeast Asian diets—including one associated with a *Blue Zone* population (7, 8) omit dairy (9). While categorizable as an omission relative to the HEI construct, these diets in fact never included dairy historically, and only occasionally do so now as elements of the Western diet are globalized. The long-standing inclusion of dairy by select populations, and its exclusion by others, has resulted in marked, demographic variation in the prevalence of lactose tolerance (10). The native, mammalian condition is lactose intolerance after infancy/weaning, and persistence of lactose tolerance throughout the lifespan represents an adaptation by certain human populations (10, 11).

Along with select, traditional Asian diets, vegan diets also exclude dairy. The traditional Paleo diet excludes dairy, and in many applications excludes grains as well (12, 13). Other diet types, whether for disease management, food intolerance, or personal preference, may exclude select food groups such as meat, poultry, fish, dairy products, and/or grain products. While not all of this impact HEI scores, some of them do.

Across a vast expanse of relevant evidence, there is no indication that health outcomes, including the most definitive—vitality and longevity—are adversely affected by the exclusion of dairy when the overall balance of the diet is sound (14). This is certainly true of meat as well, and the same is likely true for the exclusion of grains, although less evidence and fewer real-world examples pertain here. High quality versions of select Asian diets, vegan diets, and potentially Paleo diets are reasonable contenders when dietary patterns “best” for health are under consideration (15–17).

The USA is a multicultural society with a wide range of dietary practices, many based on heritage (18, 19), and others based on alternative nutrition principles and emphasis (e.g., restricting total carbohydrate intake). While routinely applied in this context, the standard application of the HEI may be ill-suited to score diets fairly across this expanse of practices. To address this limitation, and generalize the utility of routine diet quality scoring with a common metric, we introduce a simple adaptation of the HEI.

## Methods

To adapt the HEI to dietary patterns that exclude select food groups, an initial determination needed to be made about food groups that could reasonably be deemed “discretionary” in balanced, complete, and sustaining dietary patterns. The determination of “*discretionary” versus universal food components* was made by consensus of the authors, two registered dietitian nutritionists, and one physician expert in nutrition. That consensus was in turn predicated on: (1) work related to mapping the range of dietary patterns currently prevailing in the U.S.A., and to some extent other regions around the world (20); (2) the range of eating patterns represented in worldwide dietary guidelines (21) and clinical practice guidelines (22); (3) the range of dietary patterns saliently associated with longevity and health span (14, 15, 18, 23); and (4) the range of natively adapted human dietary practices (24). Across this breadth of considerations, fruits, vegetables, nuts and seeds were universal; meat, seafood, dairy, grains, and legumes were discretionary. Of note, the characterization of a given food group as discretionary depends partly on other elements in a given dietary pattern. As an example, legumes may be discretionary in a Paleolithic diet that includes meat, seafood, and/or fish, but would not be discretionary in a vegan diet excluding these alternative protein sources. Adaptations were made to the standard HEI scoring construct (1) as shown in Table 1.

**Table 1:** Scoring components: HEI-2020 vs. ACS.

Component	HEI^1^ 2020 (maximum points)	ACS^2^ (maximum points)
Adequacy		
Total fruits	5	5
Whole fruits	5	5
Total vegetables	5	5
Greens and beans	5	5
Whole grains	10	10 (optional)
Dairy^3^	10	10 (optional)
Total protein foods	5	5
Seafood and plant proteins	5	5
Fatty acids	10	10
Moderation		
Refined grains	10	10 (optional)
Sodium	10	10
Added sugars	10	10
Fatty acids	10	10

1 Healthy Eating Index.

2 Adaptive Component Scoring.

3 Includes fortified soy beverages.

The approach to Adaptive Component Scoring was developed to adjust the HEI denominator based on the food groups available to contribute “credit” to the numerator. To create the adapted formula, terms and categories were established as shown in Table 2.

**Table 2:** Established terms and categories for ACS.

Term	Categories
Total components (foods and nutrients) in the HEI score	Whole fruits; total fruits; total proteins; seafood & plant protein; greens/beans & total vegetables; nutrient entries (i.e., sat fat; added sugar; sodium; fatty acid ratio [(PUFA+MUFA)/SFA]); dairy^1^; whole grains; refined grains; total protein
Universal (required) components in the adapted HEI score	whole fruits; total fruits; seafood & plant protein; greens/beans & total vegetables; nutrient entries (i.e., sat fat; added sugar; sodium; fatty acid ratio [(PUFA+MUFA)/SFA])
Discretionary (optional) components in the adapted HEI score	dairy^1^; whole grains; refined grains; (total protein—seafood & plant protein)

1 Includes fortified soy beverages.

**Figure 1:**
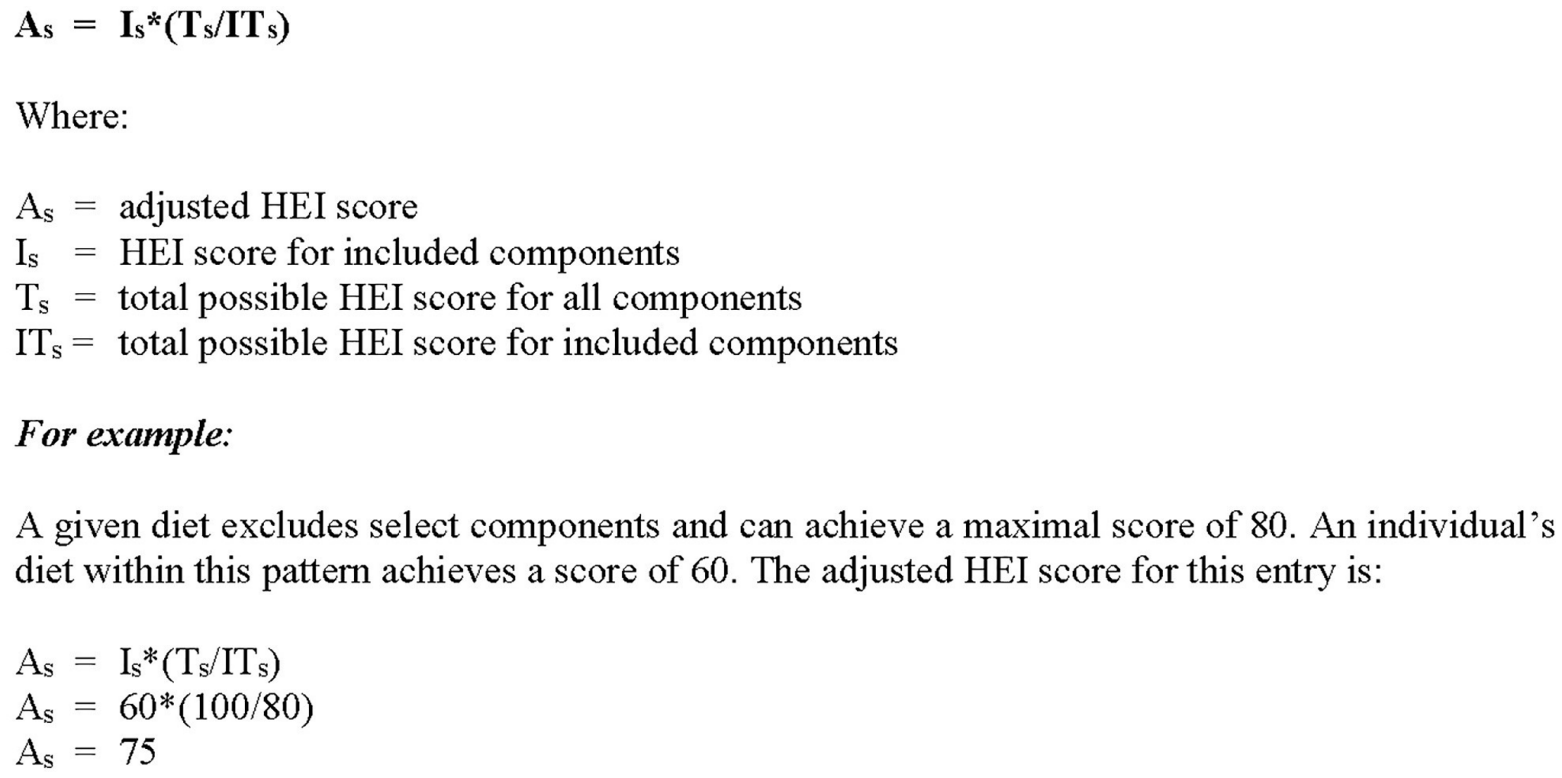
Formula for Adaptive Component Scoring.

For any given diet, the adjusted scores may be established based on the *a priori* exclusion of discretionary components, e.g., Asian diets may exclude dairy; Paleo diets may exclude dairy, grains and legumes. See Figure 1 for the adapted formula.

## Results

In practice, HEI scoring allows for full protein credit from a range of sources not excluded collectively from any balanced diet, namely: meat, poultry, fish and seafood, and plants (i.e., legumes). Thus, no diet identified required adjustment in this area. A number of diets defined by both cultural parameters and nutritional parameters require adjustment for dairy. See examples in Table 3. Select expressions of certain diets, notably Paleo and low-carb, require adjustment for grains. The maximum HEI score that can be achieved is 90 due to no credit for whole grains. See Table 3 for sample score adjustments.

**Table 3:** A representative sampling of Healthy Eating Index 2020 scores, with and without Adaptive Component Scoring applied, for optimized versions (i.e., highest achievable HEI score) of select dietary patterns that exclude one or more discretionary food groups.

Dietary Pattern	Components	Excluded Food Groups	HEI-2020 score for optimized version, unadjusted (Top Tier)	HEI-2020 score for optimized version, Adaptive Component Scoring applied
Keto	Total Fruit, Whole Fruit, Total Protein Foods, Total Vegetables, Greens and Beans, Dairy, Seafood and Plant Proteins, Fatty Acids, Refined Grains, Sodium, Added Sugars, Saturated Fat	Whole Grains	74	85
Low-Carb	Total Fruit, Whole Fruit, Total Protein Foods, Total Vegetables, Greens and Beans, Dairy, Seafood and Plant Proteins, Fatty Acids, Refined Grains, Sodium, Added Sugars, Saturated Fat	Whole Grains	89	99
Paleo, with limited non-dairy	Total Fruit, Whole Fruit, Total Protein Foods, Total Vegetables, Greens and Beans, Dairy, Seafood and Plant Proteins, Fatty Acids, Refined Grains, Sodium, Added Sugars, Saturated Fat	Whole Grains	88	97
Paleo, without dairy or dairy alternatives	Total Fruit, Whole Fruit, Total Protein Foods, Total Vegetables, Greens and Beans, Seafood and Plant Proteins, Fatty Acids, Refined Grains, Sodium, Added Sugars, Saturated Fat	Dairy, Whole Grains	80	100
Vegan	Total Fruit, Whole Fruit, Total Protein Foods, Total Vegetables, Greens and Beans, Whole Grains, Seafood and Plant Proteins, Fatty Acids, Refined Grains, Sodium, Added Sugars, Saturated Fat	Dairy	90	100
Vietnamese	Total Fruit, Whole Fruit, Total Protein Foods, Total Vegetables, Greens and Beans, Whole Grains, Seafood and Plant Proteins, Fatty Acids, Refined Grains, Sodium, Added Sugars, Saturated Fat	Dairy	89	99

When stratifying dietary patterns into 10 evenly spaced tiers (deciles) using the HEI-2020, application of Adaptive Component Scoring elevated the scores of the higher tiers for diets excluding dairy and/or grains (see Table 3). This enabled the formulation of an “optimal” diet quality tier for various East Asian diets, and high-fidelity versions of the Paleolithic diet, comparable to scores for dietary patterns with all food groups represented. Absent use of Adaptive Component Scoring, a range of cultural diets, some expressly associated with optimal health outcomes, could not achieve optimal HEI scores.

## Discussion

The quality of a given dietary pattern derives from the quality of health effects it imparts: disease prevention; health promotion; contributions to vitality and longevity. (N.B. Contributions to planetary health are of noteworthy importance, but beyond the scope of the current focus) Invoking such considerations, there is more than one way to achieve a “high quality” diet (15, 19, 25), and no one culture owns a monopoly on the formula. A universally applicable standard for high diet quality predicated on key health outcomes must allow for cultural variations, including the exclusion of a food group that has a traditional place in some cultures, but not others. Adaptive Component Scoring respects the fundamental construct of the Healthy Eating Index, while making this crucial accommodation for cultural variations.

Some food groups are clearly discretionary. There are entire human populations that have no long-standing tradition of dairying, for instance, in which lactose intolerance and the exclusion of dairy from the cultural diet both prevail. There are other populations with long exposure to dairy, and obvious adaptation to it as indicated by widespread lactose tolerance, courtesy of a genetic mutation (26). Of note, both of these groups are represented among the world’s Blue Zones, famous for their healthy life span (25, 27). This salient example illustrates the potential to achieve the same high quality of overall dietary pattern with, and without, dairy. The simple adaptation of the HEI introduced here serves as a quantitative translation of that important principle.

While there are food groups that may be deemed “discretionary” based on modern science, evolutionary biology, and the range of cultural practices, there are clearly food groups that are not. While short-term adjustments might allow for the exclusion of vegetables, fruits, or legumes from the diet, there is no discernible signal across the expanse of evidence sources noted above that such patterns are conducive to optimal health outcomes across the human lifespan. Adaptive Component Scoring was thus directed at those components of an overall dietary pattern that both (a) actually do come and go across an expanse of cultural diversity and prevailing behavior; and (b) can reliably be associated with the same set of health outcomes, summarized as years in life (i.e., longevity), and life in years (i.e., vitality). In practice, this directs the adjustments preferentially to dairy and grains. There is no need to make adjustments for the exclusion of meat, poultry, fish, or seafood, not because these do not occur, but because the HEI already accounts for this by allowing for full credit from plant-derived protein sufficient in quantity and quality (17).

Unadjusted, the HEI can present challenges when applied to dietary patterns that exclude dairy (and/or fortified soy products, which also allow quality points in the HEI) or minimize grain consumption, even if those diets are otherwise nutrient-dense and aligned with health outcomes. While it offers a robust framework for assessing diet quality, it may not fully account for dietary variations that limit these food groups, despite evidence that such diets can still promote optimal health and reduce disease risk. Several alternative scoring systems, including the Mediterranean Diet Score [MDS] (28), Alternative Healthy Eating Index [AHEI] (2), and Plant-Based Diet Index [PDI] (29), allow for reduced or absent intake of dairy or grains while still achieving high scores. These systems acknowledge that diets rich in vegetables, fruits, legumes, nuts, and healthy fats—without necessarily relying on grains or dairy—can still reduce the risk of chronic diseases such as cardiovascular disease and cancer (30, 31). However, each of these metrics is ‘fixed’ rather than adaptive in response to intrinsic dietary variation. The signature distinction of Adaptive Component Scoring is that it is, indeed, “adaptive,” giving credit as it’s due for those food components contributing to overall diet quality. As an example, whole grains are an important contributor to high-quality flexitarian diets, but may be omitted entirely from select expressions of a high-quality Paleolithic diet. Dairy is a signature element in the DASH diet, but is absent from the traditional Okinawan diet.

Other types of scoring systems, such as the Dietary Inflammatory Index [DII] (32) and NOVA classification [NOVA] (33), focus on the processing and inflammatory potential of foods rather than specific food groups, reflecting a more global, multi-cultural perspective. These approaches, too, are fixed, and not directly responsive to variation in the sources of key dietary inputs. This highlights the value of developing adaptive scoring methods that better accommodate diverse dietary patterns, including those that exclude or minimize dairy or grains, without compromising the ability to measure diet quality across various cultural and nutritional styles. Such flexibility can enhance inclusivity while maintaining the strengths of established tools like the HEI.

Attention to the diverse means of elevating overall dietary quality for a multicultural society is increasing, but has historically been limited (34). Among the important implications of this focus is the opportunity to standardize diet quality without standardizing diet type in intervention studies and food-as-medicine initiatives. The food-as-medicine movement (35) is directed to the level of population, and in particular, to population groups that are most food—and nutrition-insecure. Such groups are particularly multicultural. Familiarity is well established as a key driver of dietary preference, and adherence to prescribed diets predicated on a “one-size-fits-all” approach for a diverse population is known to be rate-limiting in their impacts; long-term adherence is a particular limitation (36).

An adaptation of the HEI for multicultural deployments offers the promise of innovations in nutrition research and service that could reduce attrition, enhance adherence, improve satisfaction, and generalize far more readily. In the food-as-medicine movement, efforts directed at the improvement of health outcomes by means of improved diet quality call for routine and standardized measurement of what is being managed. For diet quality assessment to be practiced fairly across such an expanse, it must be adapted to diverse, cultural patterns of dietary intake.

As an example of application of ACS under real-world conditions, our work involves both assessing current diet (habitual intake, rather than per-day intake), and providing guidance toward a personalized goal diet. The “improvement” in diet both intended, and achieved, is measured by change in HEI score—both for individuals, and the population. This, in turn, requires the attachment of HEI scores to goal diets. As our work involves culturally diverse populations, the personalization of goal diets also involves a multicultural array of dietary patterns. Empirically, we observed that optimized versions of select culturally diets, such as various Asian diets that omit dairy, garnered lower HEI scores than comparably, wholesome dietary patterns in other cultural lanes, inclusive of all food groups. We apply ACS when exclusion of a given HEI food group pertains because of high-fidelity adherence to a dietary type that omits that food group, generally at the higher levels (e.g., top 3 deciles) of the HEI scoring range. Application of ACS in this context serves as intended to “level the playing field,” generating comparable quality (HEI) scores for multicultural goal diets satisfying comparable nutritional parameters, while varying slightly in the food groups from which such nutrients are derived. The application of ACS to current dietary intake, along with dietary goal-setting, correspondingly pertains when (a) measurement is of habitual, not per-day, intake; and (b) that same high-fidelity adherence to optimized (i.e., upper HEI deciles) has been achieved.

We note that the generation of HEI scores predicated on dietary intake assessment presupposes, and indeed requires, that the dietary intake assessment methods applied are reliable, valid, and pertain to habitual rather than episodic intake. The same constraints pertain to the application of ACS, for which the generation of HEI scores is prerequisite.

Objective measures of diet quality are useful at both the individual and population level in risk stratification (2); in translating risk into projected costs (37); and in gauging the progress achieved in any given clinical nutrition or food-as-medicine intervention (38). Diet quality, measured objectively, has been cited as the single leading predictor variable for total chronic disease risk and premature death in developed countries around the world (5), with notable attention to that association in the United States (39). Change in overall diet quality, using a standard measure, is a useful outcome measure in nutrition research (40). Finally, overall diet quality is an important parameter to consider for both individuals and populations when establishing dietary goals. The application of ACS expands the array of dietary patterns that can meet or achieve a given quality threshold, thus expanding opportunities to tailor nutrition prescriptions to culture and native preference and measure diet quality improvement in both individuals and populations across an expanse of cultural diversity.

The introduction of Adaptive Component Scoring is intended to advance such objectives. The utility of this innovation will best be tested and affirmed in just such context.

## Data Availability

The datasets presented in this article are not readily available because use of the ACS is intended for the public domain, and pertinent data sets are accessible via public access to the Health Eating Index 2020 scoring construct. Requests to access the datasets should be directed to Lauren Q. Rhee, lauren.rhee@jointangelo.com.

## References

[1] Shams-White MM Pannucci TE Lerman JL Herrick KA Zimmer M Meyers Mathieu K. Healthy Eating Index-2020: review and update process to reflect the Dietary Guidelines for Americans, 2020-2025. J Acad Nutr Diet. (2023) 123:1280–8. doi: 10.1016/j.jand.2023.05.015, PMID: 37201748 PMC10524328

[2] Chiuve SE Fung TT Rimm EB Hu FB McCullough ML Wang M. Alternative dietary indices both strongly predict risk of chronic disease. J Nutr. (2012) 142:1009–18. doi: 10.3945/jn.111.157222, PMID: 22513989 PMC3738221

[3] Shan Z Li Y Baden MY Bhupathiraju SN Wang DD Sun Q. Association between healthy eating patterns and risk of cardiovascular disease. JAMA Intern Med. (2020) 180:1090–100. doi: 10.1001/jamainternmed.2020.2176, PMID: 32539102 PMC7296454

[4] Wang DD Li Y Afshin A Springmann M Mozaffarian D Stampfer MJ. Global improvement in dietary quality could lead to substantial reduction in premature death. J Nutr. (2019) 149:1065–74. doi: 10.1093/jn/nxz010, PMID: 31049577 PMC6543201

[5] GBD 2017 Diet Collaborators. Health effects of dietary risks in 195 countries, 1990-2017: a systematic analysis for the Global Burden of Disease study 2017. Lancet. (2019) 393:1958–72. doi: 10.1016/S0140-6736(19)30041-8, PMID: 30954305 PMC6899507

[6] Dietary Guidelines for Americans (2020). Available at: https://www.dietaryguidelines.gov/ (Accessed September 23, 2024).

[7] Huang Y Mark JG. Identification of a Blue Zone in a typical Chinese longevity region. Int J Environ Res Public Health. (2017) 14:571. doi: 10.3390/ijerph14060571, PMID: 28555035 PMC5486257

[8] Okinawa, Japan (2024). Secrets of the world's longest-living women. Available at: https://www.bluezones.com/explorations/okinawa-japan/. (Accessed September 23, 2024).

[9] Asian Heritage Diet (2024). Oldways. Available at: https://oldwayspt.org/traditional-diets/asian-heritage-diet (Accessed September 23, 2024).

[10] Tsai PM Duggan C. Malabsorption syndromes: nutritional management In: Benjamin Caballero, editor. Encyclopedia of human nutrition. Third ed. San Diego, CA: Elsevier Academic Press (2013). 136–42. doi: 10.1016/B978-0-12-375083-9.00178-1

[11] Kaufman EJ Tan C. White as milk: biocentric bias in the framing of lactose intolerance and lactase persistence. Sociol Health Illn. (2022) 44:1533–50. doi: 10.1111/1467-9566.13528, PMID: 36018892

[12] Singh A Singh D. The Paleolithic diet. Cureus. (2023) 5:e34214. doi: 10.7759/cureus.34214, PMID: 36843707 PMC9957574

[13] Harvard School of Public Health (2024). The Nutrition Source. Diet review: Paleo diet for weight loss. Available at: https://nutritionsource.hsph.harvard.edu/healthy-weight/diet-reviews/paleo-diet/ (Accessed September 23, 2024).

[14] Katz DL Friedman RS Essel KD Joshi S Levitt J. Nutrition in clinical practice (2022). Available at: https://advisor.lwwhealthlibrary.com/book.aspx?bookid=3126&sectionid=0 (Accessed September 23, 2024).

[15] Katz DL Meller S. Can we say what diet is best for health? Annu Rev Public Health. (2014) 35:83–103. doi: 10.1146/annurev-publhealth-032013-182351, PMID: 24641555

[16] Hess JM Comeau ME. Application of dairy-free vegetarian and vegan USDA food pattern models for non-pregnant, non-lactating healthy adults. J Food Sci. (2022) 87:4703–13. doi: 10.1111/1750-3841.16314, PMID: 36102227 PMC9826242

[17] Hertzler SR Lieblein-Boff JC Weiler M Allgeier C. Plant proteins: assessing their nutritional quality and effects on health and physical function. Nutrients. (2020) 12:3704. doi: 10.3390/nu12123704, PMID: 33266120 PMC7760812

[18] LeBlanc KE Baer-Sinnott S Lancaster KJ Campos H Lau KHK Tucker KL. Perspective: beyond the Mediterranean diet-exploring Latin American, Asian, and African Heritage Diets as cultural models of healthy eating. Adv Nutr. (2024) 15:100221. doi: 10.1016/j.advnut.2024.100221, PMID: 38604411 PMC11087705

[19] Traditional Diets (2024). Oldways. Available at: https://oldwayspt.org/explore-heritage-diets/why-traditional-diets/ (Accessed September 23, 2024).

[20] Katz DL Rhee L. (2022). Diet mapping processes and systems to optimize diet quality and/or minimize environmental impact. (U.S. patent number: 11328810). U.S. Patent and Trademark Office.

[21] Cámara M Giner RM González-Fandos E López-García E Mañes J Portillo MP. Food-based dietary guidelines around the world: a comparative analysis to update AESAN Scientific Committee dietary recommendations. Nutrients. (2021) 13:3131. doi: 10.3390/nu13093131, PMID: 34579007 PMC8471688

[22] Cara KC Goldman DM Kollman BK Amato SS Tull MD Karlsen MC. Commonalities among dietary recommendations from 2010 to 2021 clinical practice guidelines: a meta-epidemiological study from the American College of Lifestyle Medicine. Adv Nutr. (2023) 14:500–15. doi: 10.1016/j.advnut.2023.03.007, PMID: 36940903 PMC10201822

[23] Buettner D. (2017). The Blue Zones solution. Eating and Living Like the World’s Healthiest People. National Geographic. Washington, D.C.

[24] De La OV Zazpe I Martínez JA Santiago S Carlos S Zulet MÁ. Scoping review of Paleolithic dietary patterns: a definition proposal. Nutr Res Rev. (2021) 34:78–106. doi: 10.1017/S0954422420000153, PMID: 32482184

[25] Blue Zones (2024). Food Guidelines. Available at: https://www.bluezones.com/recipes/food-guidelines/ (Accessed September 23, 2024).

[26] Comerford KB Pasin G. Gene-dairy food interactions and health outcomes: a review of nutrigenetic studies. Nutrients. (2017) 9:710. doi: 10.3390/nu9070710, PMID: 28684688 PMC5537825

[27] Buettner D Skemp S. Blue Zones: lessons from the world's longest lived. Am J Lifestyle Med. (2016) 10:318–21. doi: 10.1177/1559827616637066, PMID: 30202288 PMC6125071

[28] Trichopoulou A Costacou T Bamia C Trichopoulos D. Adherence to a Mediterranean diet and survival in a Greek population. N Engl J Med. (2003) 348:2599–608. doi: 10.1056/NEJMoa025039, PMID: 12826634

[29] Satija A Bhupathiraju SN Rimm EB Spiegelman D Chiuve SE Borgi L. Plant-based dietary patterns and incidence of type 2 diabetes in US men and women: results from three prospective cohort studies. PLoS Med. (2016) 13:e1002039. doi: 10.1371/journal.pmed.1002039, PMID: 27299701 PMC4907448

[30] Schwingshackl L Hoffmann G Kalle-Uhlmann T Arregui M Buijsse B Boeing H. Fruit and vegetable consumption and changes in anthropometric variables in adult populations: a systematic review and meta-analysis of prospective cohort studies. PLoS One. (2015) 10:e0140846. doi: 10.1371/journal.pone.0140846, PMID: 26474158 PMC4608571

[31] Satija A Bhupathiraju SN Spiegelman D Chiuve SE Manson JAE Willett W. Healthful and unhealthful plant-based diets and the risk of coronary heart disease in U.S. adults. J Am Coll Cardiol. (2017) 70:411–22. doi: 10.1016/j.jacc.2017.05.047, PMID: 28728684 PMC5555375

[32] Shivappa N Steck SE Hurley TG Hussey JR Hébert JR. Designing and developing a literature-derived, population-based dietary inflammatory index. Public Health Nutr. (2014) 17:1689–96. doi: 10.1017/S1368980013002115, PMID: 23941862 PMC3925198

[33] Monteiro CA Cannon G Levy RB Moubarac JC Louzada ML Rauber F. Ultra-processed foods: what they are and how to identify them. Public Health Nutr. (2019) 22:936–41. doi: 10.1017/S1368980018003762, PMID: 30744710 PMC10260459

[34] Krishna P. (2020). Is American dietetics a white-bread world? These dietitians think so. The New York Times. Available at: https://www.nytimes.com/2020/12/07/dining/dietitian-diversity.html. (Accessed September 23, 2024).

[35] Food is Medicine: Current Initiative (2024). The Rockefeller Foundation. Available at: https://www.rockefellerfoundation.org/initiative/food-is-medicine/ (Accessed September 23, 2024).

[36] Vilaro MJ Staub D Xu C Mathews AE. Theory-based interventions for long-term adherence to improvements in diet quality: an in-depth review. Am J Lifestyle Med. (2016) 10:369–76. doi: 10.1177/1559827616661690, PMID: 30202295 PMC6124976

[37] Katz DL Govani R Anderson K Rhee LQ Aronson DL. The financial case for food as medicine: introduction of a ROI calculator. Am J Health Promot. (2022) 36:768–71. doi: 10.1177/08901171211070751, PMID: 35038266

[38] Vadiveloo M Lichtenstein AH Anderson C Aspry K Foraker R Griggs S. Rapid diet assessment screening tools for cardiovascular disease risk reduction across healthcare settings: a scientific statement from the American Heart Association. Circ Cardiovasc Qual Outcomes. (2020) 13:e000094. doi: 10.1161/HCQ.0000000000000094, PMID: 32762254

[39] Mozaffarian D Glickman D. (2019). Opinion: our food is killing too many of us. The New York Times. Available at: https://www.nytimes.com/2019/08/26/opinion/food-nutrition-health-care.html (Accessed December 30, 2024).

[40] McAuley EA MacLaughlin HL Hannan-Jones MT King N Ross LJ. Effectiveness of diet quality indices in measuring a change in diet quality over time: a systematic review and meta-analysis of randomized controlled trials. Nutr Rev. (2023) 81:361–83. doi: 10.1093/nutrit/nuac063, PMID: 36102824

